# An LQT2-related mutation in the voltage-sensing domain is involved in switching the gating polarity of hERG

**DOI:** 10.1186/s12915-024-01833-0

**Published:** 2024-02-05

**Authors:** Zhipei Liu, Feng Wang, Hui Yuan, Fuyun Tian, Chuanyan Yang, Fei Hu, Yiyao Liu, Meiqin Tang, Meixuan Ping, Chunlan Kang, Ting Luo, Guimei Yang, Mei Hu, Zhaobing Gao, Ping Li

**Affiliations:** 1 School of Pharmacy, Zunyi Medical University, Zunyi, 563000 China; 2 Zhongshan Institute for Drug Discovery, Zhongshan, 528400 China; 3 Center for Neurological and Psychiatric Research and Drug Discovery, Shanghai Institute of Materia Medica, Chinese Academy of Sciences, Shanghai, 201203 China; 4 School of Basic Medicine and Clinical Pharmacy, China Pharmaceutical University, Nanjing, 210009 China; 5 School of Pharmaceutical Sciences, Southern Medical University, Guangzhou, 510515 China; 6 University of Chinese Academy of Sciences, Beijing, 100049 China; 7 School of Pharmaceutical Sciences, Guizhou Medical University, Guiyang, 550025 China; 8 Pharmacology Laboratory, Zhongshan Traditional Chinese Medicine Hospital, Guangzhou University of Chinese Medicine, Zhongshan, 528401 China

**Keywords:** hERG, K525N, Gating Polarity, Voltage-Sensing Domain, LQT2

## Abstract

**Background:**

Cyclic Nucleotide-Binding Domain (CNBD)-family channels display distinct voltage-sensing properties despite sharing sequence and structural similarity. For example, the *human Ether-a-go-go Related Gene* (hERG) channel and the Hyperpolarization-activated Cyclic Nucleotide-gated (HCN) channel share high amino acid sequence similarity and identical domain structures. hERG conducts outward current and is activated by positive membrane potentials (depolarization), whereas HCN conducts inward current and is activated by negative membrane potentials (hyperpolarization). The structural basis for the “opposite” voltage-sensing properties of hERG and HCN remains unknown.

**Results:**

We found the voltage-sensing domain (VSD) involves in modulating the gating polarity of hERG. We identified that a long-QT syndrome type 2-related mutation within the VSD, K525N, mediated an inwardly rectifying non-deactivating current, perturbing the channel closure, but sparing the open state and inactivated state. K525N rescued the current of a non-functional mutation in the pore helix region (F627Y) of hERG. K525N&F627Y switched hERG into a hyperpolarization-activated channel. The reactivated inward current induced by hyperpolarization mediated by K525N&F627Y can be inhibited by E-4031 and dofetilide quite well. Moreover, we report an extracellular interaction between the S1 helix and the S5-P region is crucial for modulating the gating polarity. The alanine substitution of several residues in this region (F431A, C566A, I607A, and Y611A) impaired the inward current of K525N&F627Y.

**Conclusions:**

Our data provide evidence that a potential cooperation mechanism in the extracellular vestibule of the VSD and the PD would determine the gating polarity in hERG.

**Supplementary Information:**

The online version contains supplementary material available at 10.1186/s12915-024-01833-0.

## Background

The *human Ether-a-go-go Related Gene* channel (hERG) is expressed as the rapidly activating delayed rectifier potassium current (*I*_Kr_) in cardiac myocytes, and it plays a critical role in the repolarization of the action potential (AP) of ventricular cardiomyocytes [1]. The deficiency of hERG is usually associated with long-QT syndrome type-2 (LQT2), featured by a prolongation of the QT interval of the surface electrocardiogram (ECG) and T-wave abnormalities [2, 3]. hERG is a depolarization-activated potassium channel, characterized by an extremely fast and atypical C-type inactivation [1, 4–6]. Channels will conduct potassium when hERG is in an activated state, but will not conduct ions when it is in an inactivated state and a closed state [1, 6]. Because hERG activation is slow relatively than rapid inactivation, its current-voltage (*I*-*V*) curve is demonstrated as a bell-shaped relationship [1, 6].

hERG belongs to the cyclic nucleotide-binding domain (CNBD) clade of the voltage-gated potassium channel (Kv) superfamily, which contains hyperpolarization-activated and cyclic nucleotide-gated (HCN) channels as well [7]. Both hERG and HCN channels share a similar architecture with a centrally-located pore flanked by four identical subunits [6, 8, 9]. Each channel is a homotetramer, composed of four subunits. Each subunit consists of 6 transmembrane helices (S1-S6), in which S1-S4 is the voltage-sensing domain (VSD), S5-S6 is the pore domain (PD), and the carboxy-terminal is the cyclic nucleotide-binding domain (CNBD) [6, 8, 9]. The S4-S5 linker determines the coupling between VSD and PD [1, 10, 11]. Upon depolarization, the S4 helix will translate along its axis outwardly and move across the membrane [12–15]. This gating motion of the four VSDs can drive the conformational changes in the coupling machinery of the S4-S5 linker that opens the channel’s gate eventually [11, 12, 15, 16]. Despite these structural similarities between hERG and HCN channels, hERG is activated by membrane depolarization whereas HCN channels are activated by hyperpolarization [6, 8, 9]. It is a mystery that how channel proteins with such high homology are elicited by completely different membrane voltages (depolarization vs. hyperpolarization) and mediate opposite currents. Since hERG and HCN channels have evolved from the same ancestor [7], it might be reasonable to speculate the structural basis for opposite gating polarity could be subtle and may lie in variations of several amino acid residues. However, the structural basis for the different voltage dependence between hERG and HCN channels is still largely unknown.

The PD of hERG contains all the molecular elements necessary for potassium conduction and for activation and inactivation gating. Activation is classically associated with conformational rearrangements at the inner helix bundle (the activation gate) whereas C-type inactivation involves structural changes at the selectivity filter (the inactivation gate) [1, 11, 17]. The motion of the S4 helix drives the activation gate through the S4-S5 linker at the intracellular surface and modulates the inactivation gate through the S1-S4 near the extracellular surface [15, 18–25]. Converting the depolarization-activated channel of hERG into a hyperpolarization-activated channel can be achieved by mutating a single residue in either the S4-S5 linker [23, 26] or the S6 helix [27]. D540K of hERG is a well-known channel that carries the reactivated inward current induced by hyperpolarization [23, 26]. Proline substitution of V659 (V659P) within the S6 helix of hERG also passes inward non-deactivating current upon hyperpolarization [27]. The conductance-voltage (*G*-*V*) relationship of both the D540K and V659P channel exhibits an increased conductance upon hyperpolarization, which is well described by a Boltzmann function with a *V*_1/2_ around -100 mV (-117 mV for D540K [26], and -99.7 mV for V659P [27]). For hERG and several other depolarization-activated ion channels [28, 29], the polarity of ion channel gating can be determined by the pore domain as well.

In this study, we found the voltage-sensing domain also plays a critical role in modulating the gating polarity of hERG. An LQT2-related mutant on the S4 helix, K525N [30], mediated an inwardly rectifying non-deactivating current upon hyperpolarization. K525N perturbed pore gate closure and preserved the open and inactivated state. Interestingly, K525N together with either F627Y (K525N&F627Y) or hERG blockers, created a hyperpolarization-activated channel with *V*_1/2_ is about -103.2 mV. K525N&F627Y was still inhibited by pore blockers, dofetilide, and E4031. Moreover, we found that a potential extracellular interaction between the S1 helix and the S5-P region is critical for the hyperpolarization-activation channel of K525N&F627Y.

## Results

### K525N&F627Y is a hyperpolarization-activated channel

**Fig. 1:**
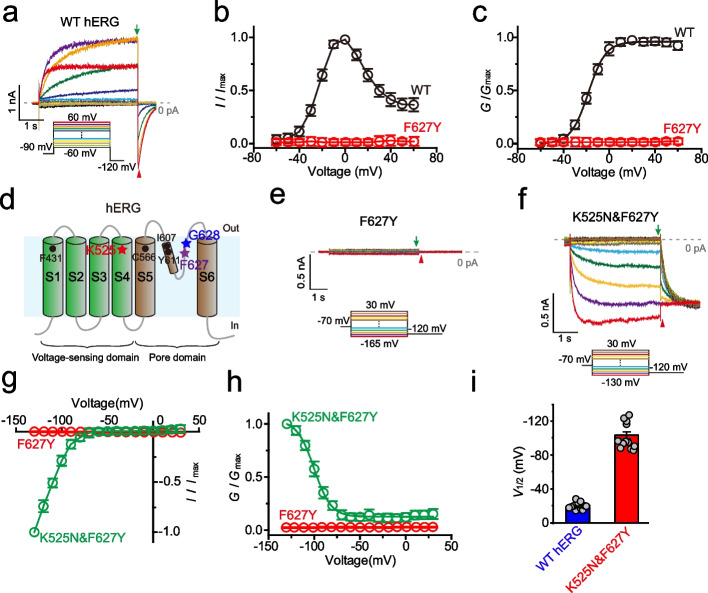
K525N&F627Y is a hyperpolarization-activated channel. **a** Representative currents of WT hERG overexpressed in CHO cells. The step voltage protocol (below) varies from -60 to +60 mV with a holding potential of -90 mV. The green arrow and red arrowhead indicate the measurement region of outward currents and tail currents, respectively. The 0 pA is indicated by a dashed line. **b** The normalized current-voltage (*I*-*V*) curve of WT hERG (n≥6) and F627Y (n≥6). Measured currents are indicated by the green arrow in Fig. 1a. **c** The normalized conductance-voltage (*G*-*V*) curve of WT hERG (n≥6). Measured currents are indicated by the red arrowhead in Fig. 1a. **d** Diagram of an hERG channel subunit that can be divided into a VSD (S1-S4) and a PD (S5-S6). K525 in the S4 helix and F627 in the selective filter is indicated by a red and purple star, respectively. The potential interacting residues (F431, C566, I607, and Y611) are also labelled with black dots. F431 is in S1, C566 is in S5, I607 and Y611 are in the pore helix. **e** Representative currents of F627Y. Step voltage protocol (below) varies from -165 to +30 mV with a holding potential at -70 mV. **f** Representative currents of K525N&F627Y. Step voltage protocol (below) varies from -130 to +30 mV with a holding potential at -70 mV. **g** The normalized *I*-*V* curve (n≥6) and **h** The normalized *G*-*V* curve (n≥6) of K525N&F627Y. **i** *V*_1/2_ values of WT hERG and K525N&F627Y (*n*=12). Error bars represent mean ± SEM

We chose to use hERG as a model channel, to explore the role of the voltage-sensing domain in modulating its rectification property. On transfection with hERG cDNA, CHO-K1 cells expressed a large potassium current with biophysical properties reminiscent of the *I*_Kr_ current in cardiomyocytes [6] (Fig. 1a). A slowly activating potassium current developed during depolarizations above -60 mV with maximum at a potential 0 mV in hERG-expressing cells (Fig. 1b). It opens with evident inactivation at more positive potential (Fig. 1a, and b). Analysis of the tail current allowed us to characterize the half-activation voltage (*V*_1/2_) which is -18.7 ± 1.1 mV (Fig. 1c, and i). The C-type inactivation gate of hERG contains _626_GFG_628_ motif in the selectivity filter [4, 5]. As one of the key determinants of C-type inactivation, F627 regulates the asymmetrical constricted-like conformation of the selectivity filter [4] (Fig. 1d). The tyrosine substitution of F627 (F627Y) leads to an ion non-conducting channel under physiology solutions (Fig. 1a-e), consistent with previous studies [4, 31–33].

We investigated channel properties of many double mutants that combined mutations of gating charge and F627Y (Fig. 1d). To our surprise, the nonfunctional single mutant of F627Y was rescued by the K525N mutation (Fig. 1f). Moreover, K525N&F627Y was activated by membrane hyperpolarization and mediated non-deactivated inward current (Fig. 1f-h, and Additional file 1: Fig. S1), in stark contrast to the wild-type (WT) hERG that was activated by membrane depolarization and conducted outward current (Fig. 1a, and b). Such behavior is reminiscent of the hyperpolarization-induced activation of D540K [23, 26] and V659P [27] of hERG, W434F-P475D of Shaker-IR [34], L226P of NachBac [28], and L271P&D312N of SKOR [29]. We observed K525N&F627Y was completely closed at physiological membrane potentials and began to conduct a slowly activated inward current as the membrane voltage was changed from -70 to -135 mV (Fig. 1f, g, and Additional file 1: Fig. S1). Such activation properties are exactly opposite to those of WT hERG, which was activated by more positive membrane potentials (Figs. 1a, and b; from -60 to +60 mV). The voltage dependence of the peak tail current for K525N&F627Y was fitted to a Boltzmann function, yielding a *V*_1/2_ of -103.2 ± 4.1 mV (Fig. 1h, and i), 84.5 mV more negative than that for WT (Fig. 1i). Also, K525N&G628S is a hyperpolarization-activated channel similar with K525N&F627Y (Additional file 1: Fig. S1). The gating polarity of K525N&F627Y and K525N&G628S is reversed to WT hERG (Fig. 1c, h, and Additional file 1: Fig. S1).

**Fig. 2:**
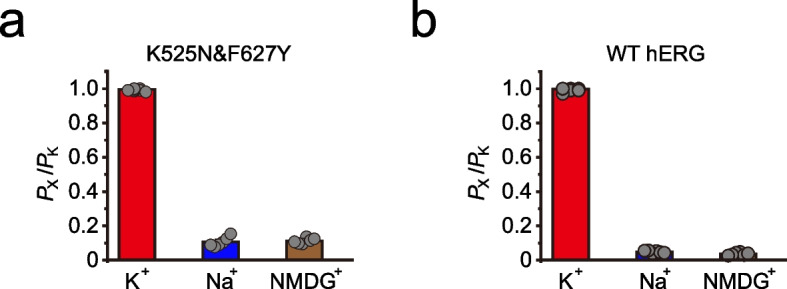
K525N&F627Y is a K^+^ selective channel. **a** Analysis of relative K^+^ permeability over Na^+^, or NMDG^+^ of K525N&F627Y based on *E*rev measurement (n≥6). **b** Analysis of relative K^+^ permeability over Na^+^, or NMDG^+^ of WT hERG based on *E*rev measurement (n≥6). Error bars represent mean ± SEM

Under physiological solutions (external: 5 mM K^+^ and 140 Na^+^ versus internal: 145 mM K^+^), the reversal potential of WT hERG and K525N&F627Y was around -60 mV (Additional file 2: Fig. S2); and it changed to about 0 mV (Additional file 2: Fig. S2) while symmetric potassium solutions were applied (external and internal: 145 mM K^+^). Therefore, the reversal potential of WT hERG and K525N&F627Y was strictly determined by the potassium gradient between the extracellular (external) solution and intracellular (internal) solution. K525N&F627Y and K525N&G628S remained a potassium channel with a limited permeability to sodium and NMDG (Figs. 2a, and Additional file 2: Fig. S2), which is similar to WT hERG (Fig. 2b).

### K525N&F627Y is inhibited by hERG channel blockers

**Fig. 3:**
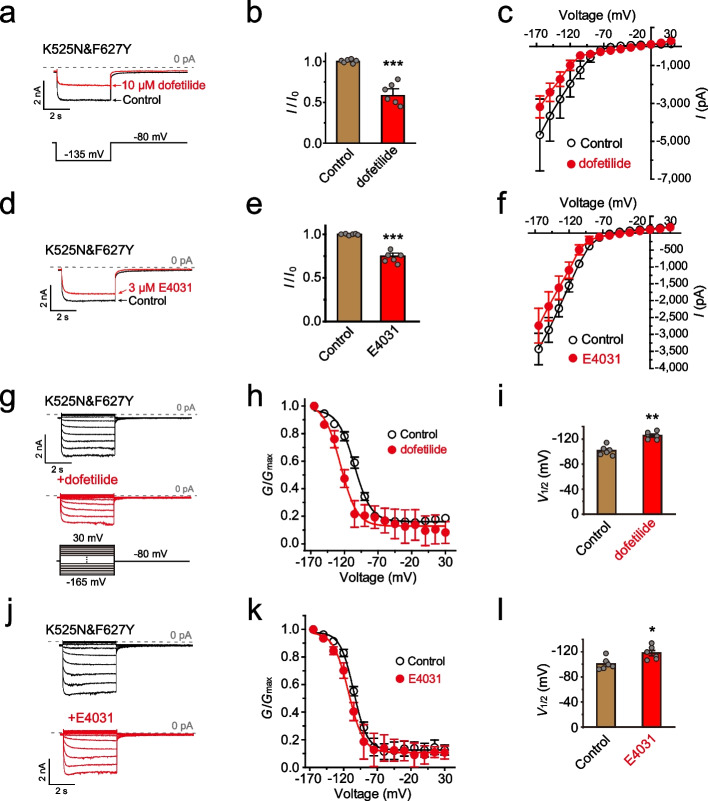
K525N&F627Y is inhibited by dofetilide and E4031. **a** Representative currents of K525N&F627Y with or without 10 μM dofetilide. Inward currents were elicited with -135 mV (the protocol see below). **b** histogram of *I*/*I*_0_ at -135mV with or without dofetilide (n≥6). **c** *I*-*V* curves of K525N&F627Y with or without dofetilide (n≥6). **d** Representative currents of K525N&F627Y with or without 3 μM E4031. **e** histogram of *I*/*I*_0_ at -135mV with or without E4031 (n≥6). **f** *I*-*V* curves of K525N&F627Y with or without E4031 (n≥6). **g** Representative step currents of K525N&F627Y with or without dofetilide. The step voltage protocol (below) varies from -165 to +30 mV. **h** The *G*-*V* curve (n≥6) and **i** *V*_1/2_ values (n≥6) of K525N&F627Y with or without dofetilide. **j** Representative step currents of K525N&F627Y with or without E4031. **k** The *G*-*V* curve (n≥6) and **l** *V*_1/2_ values (n≥6) of K525N&F627Y with or without E4031. Error bars represent mean ± SEM. *P<0.05, **P<0.01; ***P<0.001, two-tailed Student’s t-test

hERG channel physiologically conducts ions through the alpha (α) pore [6]. However, the omega (Ω) pore would be created by mutating some gating charges in the S4 of voltage-gated ion channels, especially the first gating charge [35–37]. K525S has been reported that it can create an extra Ω pore in hERG [38]. Therefore, a question is raised on whether the hyperpolarization-activated conductance of K525N&F627Y is indeed carried by the hERG channel, and if true, whether it is still carried by the α pore. To address these questions, we first investigated the K525N&F627Y current sensitivity to some hERG channel blockers. Dofetilide and E4031 are both classical blockers through sitting in the central cavity of the α pore [39–41]. We found that both dofetilide (Fig. 3a-c) and E4031 (Fig. 3d-f) also significantly inhibited the inward current of K525N&F627Y. The sensitivity of K525N&F627Y to both blocker is dramatically reduced compared with wild-type hERG, which might be attributed to an inability of the open gate to trap blockers in the pore at hyperpolarized potentials [40]. The similar phenomenon was also observed for the D540K mutant [40]. The *G*-*V* curve of K525N&F627Y was dramatically left-shifted in the presence of either dofetilide (Fig. 3g-i) or E4031 (Fig. 3j-l). Given that dofetilide and E4031 often act as blocker binding in the α pore [39, 41], and the K525N&F627Y channel shares the same ion selectivity and permeability as the WT hERG channel (Figs. 2, and Additional file 2: Fig. S2), it is reasonable to speculate the inward current of K525N&F627Y is still through the α pore as WT hERG.

### K525N perturbs the channel closed state, presents an open and an inactivated state

**Fig. 4:**
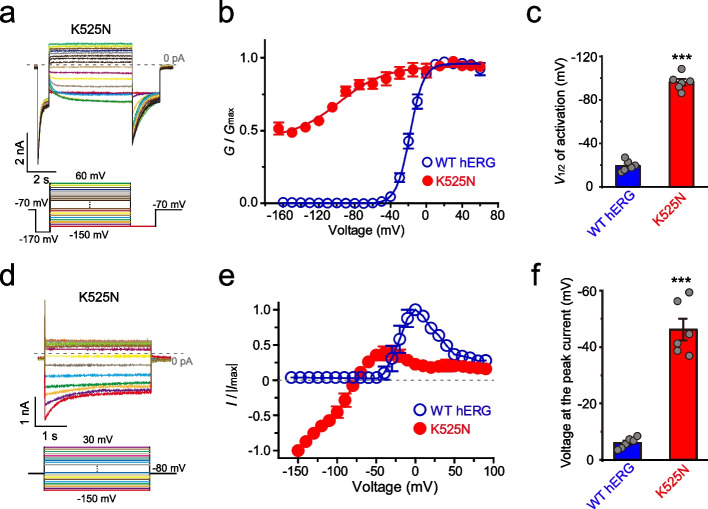
K525N perturbs the channel closure and preserves the open and inactivated state. **a** Representative step currents of K525N elicited with a complicated protocol. The step voltage protocol (below) varies from -150 to +60 mV with a holding potential of -70 mV. Since channels were instantaneously open at -70 mV, a -170 mV was applied before the step trying to close the pore gate and to detect the activation kinetics. A -150 mV followed the step to detect tail currents. **b** The *G*-*V* curve (n≥6) and **c** *V*_1/2_ of activation values (n≥6) of K525N. **d** Representative step currents of K525N elicited with an easier protocol. The step voltage protocol (below) varies from -150 to +60 mV. **e** The *G*-*V* curve (n≥6) and **f** the voltage at the peak current (n≥6) of K525N. Error bars represent mean ± SEM. ***P<0.001, two-tailed Student`s t-test

To explore the hyperpolarization-activated gating mechanism of K525N&F627Y, we characterized the channel properties of K525N. K525 is proximate to the S3-S4 linker that regulates hERG activation gate [42, 43]. Unlike WT hERG, K525N produced an instantaneous current that was observed upon hyperpolarization as well as depolarization (Fig. 4a, and d). K525N still exhibited properties of a depolarization-activated channel, and tail currents at -150 mV were progressively larger as if the membrane potential was depolarized (Fig. 4a, b, and Additional file 3: Fig. S3). The tail current demonstrated compromised deactivation, and half of the maximum conductance remained at -150 mV (Fig. 4a, and b). K525N can not be fully closed even if the voltage is -170 mV (Fig. 4a, and b). The *G*-*V* curve of K525N is dramatically left-shifted compared with WT hERG (Fig. 4b), giving a *V*_1/2_ of -95.6 ± 3.9 mV and -19.3 ± 2.1 mV for K525N and WT, respectively (Fig. 4c). An inwardly rectifying current was still detected at more positive potentials (-46.2 ± 3.9 mV) with a maximum current recorded at more negative potentials than WT hERG (6.0 ± 0.8 mV) (Fig. 4d-f). With further depolarization, the current of K525N amplitude decreased progressively (Fig. 4d, and e), which is due to the channel voltage-dependent inactivation. Thus, K525N perturbed channel closure and preserved the channel activation state and inactivation state.

### The gating polarity of K525N is pharmacologically reversed by E4031 and cisapride

**Fig. 5:**
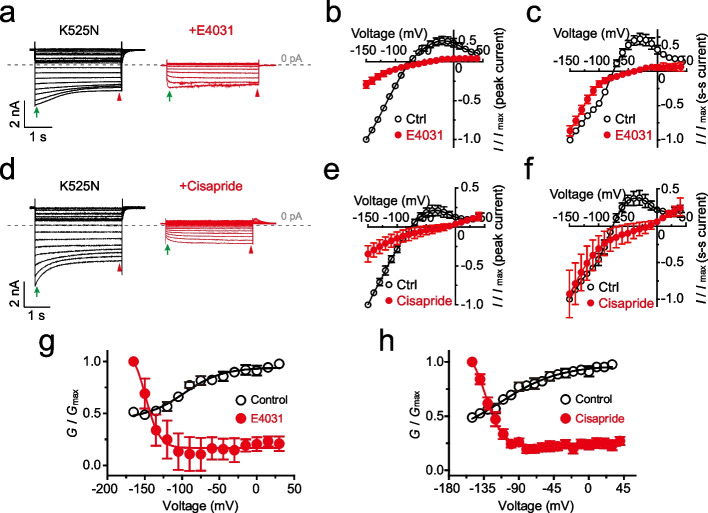
The gating polarity of K525N is switched by E4031 and cisapride. **a** Representative step currents of K525N with or without 3 μM E4031. Green arrows indicate peak currents, and red arrowheads indicate steady-state currents. **b** *I*-*V* curves of peak currents (n≥6) and **c** steady-state currents (n≥6) of K525N with or without E4031. **d** Representative step currents of K525N with or without 10 μM cisapride. Green arrows indicate peak currents, and red arrowheads indicate steady-state currents. **e** *I*-*V* curves of peak currents (n≥6) and **f** steady-state currents (n≥6) of K525N with or without cisapride. *G*-*V* curves of K525N with or without **g** E4031 and **h** cisapride. Error bars represent mean ± SEM

Both E4031 and cisapride bind in the central cavity of the PD near the inactivation gate of hERG channel [39, 41, 44]. To further explore the role of the inactivation in the gating polarity of hERG, we studied the influence of E4031 and cisapride on the channel properties of K525N. In the presence of E4031 at 3 μM (> IC_100_ = 1 μM on WT hERG in CHO cells [45]) and Cisapride at 10 μM (> IC_100_ = 1 μM on WT hERG in CHO cells [46]), the outward current was almost completely blocked (Fig. 5a, and d). However, a partial inward current of K525N remained (Fig. 5a, and d), showing the peak current was more sensitive to E4031 and cisapride than the steady-state current (Fig. 5b, c, e, and f). The residual inward current (Fig. 5a, and d) is reminiscent of that of K525N&F627Y (Fig. 1f). The tail current of the residual inward current was increased as the membrane potential decreased from -100 mV (Fig. 5g, and h). The *G*-*V* curve can be fitted by a Boltzmann function with the *V*_1/2_ of -147.0 ± 12.7 mV and -119.6 ± 5.4 mV for K525N with E4031 and cisapride, respectively (Fig. 5g, and h). E4031 and cisapride converted a depolarization-activated channel of K525N into a hyperpolarization-activated channel.

### The extracellular interaction between the VSD and PD is critical for functions of K525N&F627Y

**Fig. 6:**
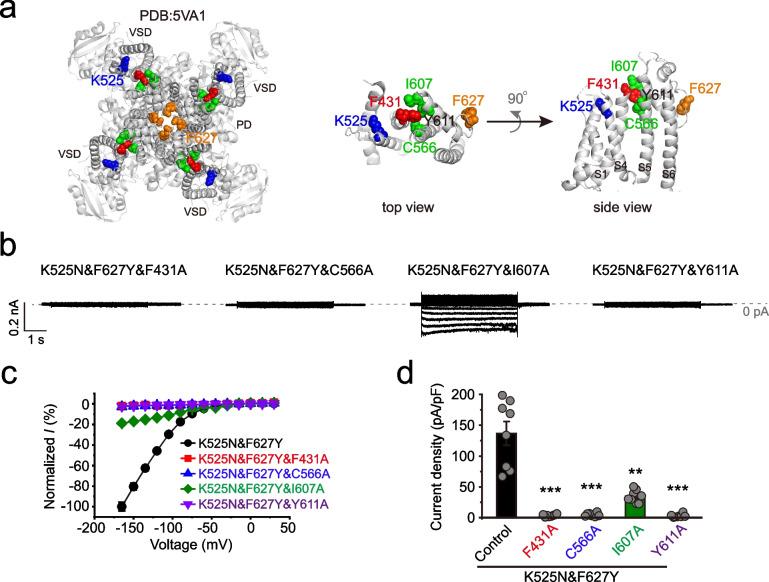
F431A, C566A, I607A, and Y611A impair the inward current of K525N&F627Y. **a** The cryo-EM structure of hERG (PDB: 5VA1) [6] highlighted with K525 (blue), F627 (yellow), and potential key residues (red in the S1 helix, and green in the S5-P region) of the extracellular interaction face between the VSD and the PD. The top view and side view of the interaction region are zoomed out and shown in the *Right* panel. **b** Representative step currents of F431A, C566A, I607A, and Y611A on top of K525N&F627Y, respectively. **c** Normalized *I*-*V* curves (n≥6) and **d** current density (n≥6) of F431A, C566A, I607A, and Y611A on top of K525N&F627Y, respectively. Error bars represent mean ± SEM. **P<0.01; ***P<0.001, two-tailed Student’s t-test

The extracellular and intracellular interaction of the voltage-sensing domain and the pore domain regulate voltage-gated ion channel functions including the hERG channel [18, 20, 22, 24, 25, 47]. The VSD-PD interaction is the mechanism that confers voltage-dependent inactivation in hERG, and whether the hyperpolarization channel of K525N&F627Y can be regulated by the extracellular interaction of the VSD and the pore is not clear. Based on the cryo-EM structure of hERG (PDB:5VA1) [6], we observed the extracellular interaction is potentially composed of the F431 on the S1, C566 in the S5, I607, and Y611 on the pore helix [6, 19, 22] (Fig. 6a). These potential interaction sites are flanked by K525 in the S4 and F627 in the selective filter (Fig. 6a). Mutating F431, C566, I607, and Y611 to the alanine with K525N&F627Y background did not change the expression pattern of hERG on the plasma membrane (Additional file 4: Fig. S4). With mutations of F431A, C566A, and Y611A, K525N can not rescue the current of F627Y at all. The mutation of I607A also partially disrupted the rescue effect of K525N on F627Y, and the inward current of K525N&F627Y&I607A was significantly reduced (Fig. 6b-d). Mutations of other residues beyond this region, such as I409A, barely affected functions of K525N&F627Y (Additional file 4: Fig. S4). F431A, C566A, and Y611A eliminated the current of K525N as well (Additional file 4: Fig. S4). Therefore, the potential interaction between the VSD and PD is also involved in the hyperpolarization-activated properties of K525N&F627Y.

### K525N&S631A is a “leak” channel

**Fig. 7:**
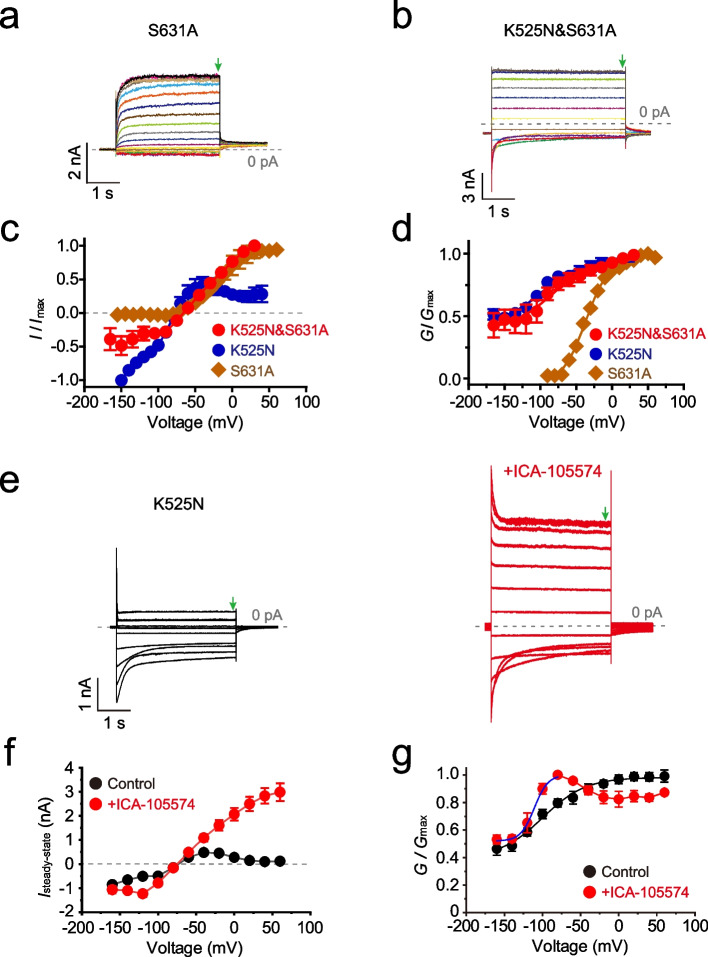
K525N&S631A is a “leak” channel. **a** Representative step currents of S631A, and **b** K525N&S631A. **c** *I*-*V* curves (n≥6) of S631A, K525N, and K525N&S631A, respectively. Steady-state currents indicated by green arrows in Fig. 7a and b were quantified. **d** *G*-*V* curves of S631A, K525N, and K525N&S631A, respectively. **e** Representative step currents and **f** *I*-*V* curves (n≥6) of K525N with or without 10 μM ICA-105574. **g** *G*-*V* curves of K525N with or without 10 μM ICA-105574. In the presence of ICA-105574, the *G*-*V* curve was fitted by the Boltzmann equation (blue line). Error bars represent mean ± SEM

Previously, S631A was reported as an “inactivation-alleviation” mutant [48, 49]. Consistently, we found that the inactivation was absent even at +50 mV in S631A [48, 49] (Fig. 7a, and c). We first combined S631A with K525N to create K525N&S631A and recorded the channel behavior at a large range of membrane potentials (Fig. 7b). Interestingly, K525N&S631A was a “leak” channel with substantial inward and outward currents (Fig. 7b, and c). The inward current of K525N&S631A was different from K525N, as a saturated current was achieved at negative potentials (Fig. 7b, and c). However, K525N&S631A is still a depolarization-activated channel with the voltage dependence similar to K525N (Fig. 7d). ICA-105574 is a potent activator of hERG by alleviating the inactivation [50–52]. In the presence of ICA-105574, K525N also behaved as a “leak” channel (Fig. 7e), and the inward current was saturated at negative potential as well (Fig. 7f). ICA-105574 left-shifted the *G*-*V* curve of K525N channel at potential more negative than -80 mV significantly (Fig. 7g). Thus, the VSD and the PD together could also reshape hERG to a “leak” channel.

## Discussion

### The VSD is involved in switching the gating polarity of hERG

Both hERG and HCN belong to CNBD-family channels, sharing a similar structure [6, 8, 9]. Recently, several studies have revealed that the structural basis for the different polarities of the depolarization-activated channel and hyperpolarization-activated channel is associated with the pore domain of hERG [23, 26, 27]. D540K in the S4-S5 linker [23, 26] and V659P in the S6 helix [27] of hERG mediate the inward current that is gradually larger as if the membrane potential decreases. K540 interacts with R665 in the S6 helix to couple the VSD with the PD, underlying the mechanism of hyperpolarization-dependent opening of D540K [23]. V659P introduces a kink that disrupts the coupling between the VSD and the PD [27]. In the Shaker channel, the combination of L434F and P475D, two residues in the PD, converts it to the hyperpolarization activated channel as well [34]. Whether the VSD is involved in determining the gating polarity of hERG? In HCN-EAG chimera channels, the substitution of serine in helix breaking transition in the S4 with a bulky hydrophobic amino acid flips the gating polarity from inward to outward-rectifying [53], suggesting the VSD may be critical to the gating polarity. The S3-S4 linker is important for stabilizing the closed conformation of hERG relative to the open state [42, 43, 54]. As the first gating charge, K525 stabilizes the closed state of hERG. Mutations of K525 would disturb the pore gate closure, and left-shift the voltage dependence dramatically [43, 54–58]. We found herein K525N also impairs the pore gate closure and preserves the open and inactivated state. When cooperating with F627Y, a mutation in the inactivation gate [4, 31, 32], K525N successfully inverted the polarity of voltage dependence (Fig. 1e-h). Therefore, the VSD is also involved in modulating the gating polarity of hERG.

### The hyperpolarization-activated mechanism of K525N&F627Y

**Fig. 8:**
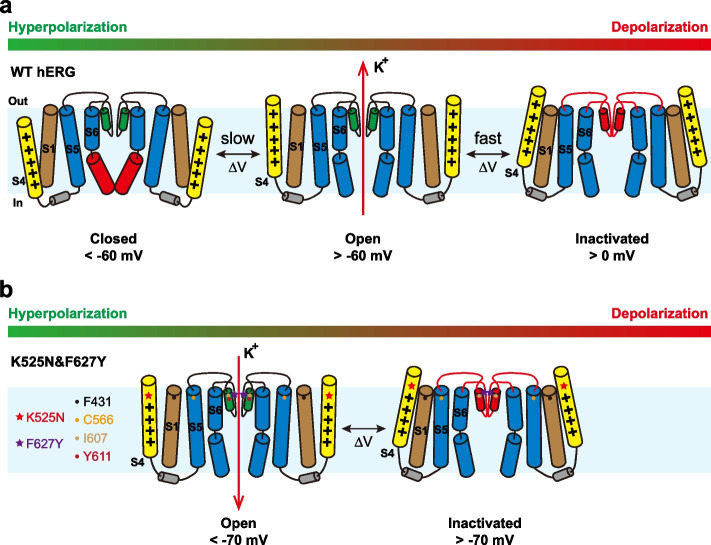
Suggested model for the inverted gating polarity of K525N&F627Y. **a** Upon depolarization, WT hERG sequentially forms three states, the closed, the open, and inactivated state. As the membrane potential depolarizes, S4 (yellow) of hERG slides extracellularly and slowly drives the activation gate open (red) via the S4-S5 linker (grey). Channels transit from a closed state into an open state that mediates an outward K^+^ current. Once the membrane potential is higher than 0 mV, S4 further translocates extracellularly, and channels are inactivated. Channels reach the inactivated state that is non-conductive to ions. The non-canonical coupling pathway between the VSD and PD is critical for the hERG channel function. **b** K525N&F627Y preserves two states of hERG, the open and the inactivated state. When the membrane potential is higher than -70 mV, S4 (yellow) usually sits extracellularly. Channels stay in the inactivated state that is non-conductive to ions. Once the membrane potential hyperpolarizes, S4 would translocate mildly toward the intracellular, and relieve the inactivation gate. Channels transit into a “leaky open” state that mediates an inward K^+^ current

The PD of the hERG channel contains the intracellular activation and extracellular inactivation gate [6]. Both gates are intimately regulated by the VSD (Fig. 8a) [6]. In the closed state at the rest membrane potential, the voltage sensor is located intracellularly, leading to the activation gate being closed and the inactivation gate being open [1, 6, 11]. Upon depolarization, the voltage sensor slides outwardly, inducing the activation gate to gradually open (Fig. 1a, and c) via the intracellular S4-S5 linker. Whereas the inactivation gate would keep open at a voltage lower than 0 mV, and close at higher voltages (Fig. 1a, and b). How the gating polarity of hERG is reversed in K525N&F627Y? K525 is the first gating charge of hERG [6], and eliminating the first gating charge may prohibit S4 from sliding down completely at hyperpolarization and prevent full closure of the activation gate [13, 59]. Since K525N left-shifts the voltage dependence significantly and eliminates the closed state of the channel (Fig. 4a, and b), the S4 might be located extracellularly, preventing full closure of the activation gate and resulting in a “leaky open” state in K525N&F627Y (Fig. 8b). The inactivation gate may still open at hyperpolarizing potentials, resembling the Shaker-IR-L434F-P475D channel [34]. However, as the membrane potential depolarizes, the inactivation gate gradually close (Fig. 8). K525N&F627Y only exhibits the open and the inactivated state without the closed state. Therefore, it is the inactivation gate that determines the ion conductance of K525N&F627Y, which is reminiscent of the Shaker-IR-L434F-P475D channel [34], and the voltage-dependent gate in the MthK potassium channel [60].

### Non-covalent interactions of the VSD and PD in Kv channels

Two tetrameric architectures identified amongst Kv channels: domain-swapped channels, such as Shaker, Kv1.2, and Kv7.1, and non-domain-swapped channels, such as hERG, HCN, and KvAP [6]. In domain-swapped channels, the VSD from one subunit is in close contact with the PD from the adjacent subunit, forming an extensive inter-subunit non-covalent interface. In non-domain-swapped channels the VSD and PD from the same subunit closely contact with a non-covalent interface as well. The non-covalent interface is located at the extracellular region of the VSD and PD. The non-covalent interface of domain-swapped channels is similar with that of non-domain-swapped channels, which is supported by the Shaker channel [20] and hERG [25]. In Shaker K^+^ channel, the voltage sensor is coupled with the selective filter (VS-SF coupling) through the extracellular interaction chain composed of the S4, S5, P-Loop, and SF [20]. The L366H in the S4 restores the K^+^ conduction abrogated by W434F in the PD of the Shaker-IR channel. The disruption of the functional connection between contiguous residues in the S4-S5-(P-Loop)-SF chain is sufficient to decrease the K^+^ conduction of L366H:W434F [20]. A kinematic chain of residues that couples the VSD to PD consists of S4/S1 and S1/S5 subunit interfaces in hERG [25]. The movements of VSD electromechanically propagates to the constriction of the SF following the S4-S1-S5-(P-Loop)-SF route relevant for the C-type inactivation [25]. These paths in hERG resemble those for the Shaker channel, suggesting the presence of a common underlying mechanism of electromechanical coupling across the superfamily of Kv channels.

### K525N and drug treatment

LQT2-associated mutations broadly distribute in every domain of hERG [2, 30, 61]. A comprehensive study analyzing 226 different LQT mutations reported that 32% resided in transmembrane and pore domains [61]. Deficient protein trafficking to the cell membrane is the dominant mechanism for most of the mutations associated with LQT2 [3, 62]. The trafficking defect mutants can be pharmacologically corrected by culturing in the presence of pore-blocking drugs such as E4031 [62, 63]. Although K525N is still functional on the cell surface (Fig. 4), many channel functional properties are different from WT hERG. Neither activators nor inhibitors of hERG can restore the functions of K525N (Figs. 5 and 7). Therefore, our study suggests alternative treatments should be developed for LQT2 patients carrying the K525N mutation. In addition, LQT2 syndrome in patients with K525N&R528P [30] may not be pathologically attributed to the K525N, since K525N is a gain-of-function mutant of hERG.

## Conclusions

This work characterized the role of a long-QT syndrome type-2-related mutation in the voltage-sensing domain, K525N [30], in modulating the gating polarity of hERG. Completely different from WT hERG, K525N together with F627Y reversed a depolarization-activated channel into a hyperpolarization-activated channel. K525N&F627Y conducted ions upon hyperpolarization and was non-conducted under depolarization. A Boltzmann fitting curve of the tail current gave a *V*_1/2_ is about -103.2 mV. The non-deactivated inward current conducted by K525N&F627Y was still sensitive to hERG channel blockers. Pharmacologically, a hyperpolarization-activated channel was also achieved by blocking K525N with E4031 and cisapride. Furthermore, we discovered that the extracellular interaction between the S1 helix and the S5-P region was crucial for the involvement of the VSD in modulating the gating polarity of hERG.

## Methods

### Mutagenesis

The hERG construct was a gift from M. Sanguinetti (University of Utah), which has been described in our previous work [64]. hERG mutants were generated with QuikChange lightning II site-directed mutagenesis kit (Agilent) and confirmed by DNA sequencing. Primers used for site-directed mutagenesis are listed below.

**Table:** 

mutation	Forward primer	Reverse primer
K525N	gatcgggctgctgaacactgcgcggctg	cagccgcgcagtgttcagcagcccgatc
F627Y	ctcaccagtgtgggctacggcaacgtctc	gagacgttgccgtagcccacactggtgag
G628S	cagtgtgggcttcagcaacgtctctcccaac	gttgggagagacgttgctgaagcccacactg
F431A	ccctactcggctgccgccctgctgaaggagac	gtctccttcagcagggcggcagccgagtaggg
C566A	cgcactggctagccgccatctggtacgccatc	gatggcgtaccagatggcggctagccagtgcg
I607A	ctgggcggcccctccgccaaggacaagtatg	catacttgtccttggcggaggggccgcccag
Y611A	cctccatcaaggacaaggctgtgacggcgctctac	gtagagcgccgtcacagccttgtccttgatggagg
I419A	catcctgctgctggtcgcctacacggctgtcttc	gaagacagccgtgtaggcgaccagcagcaggatg
S631A	gcttcggcaacgtcgctcccaacaccaac	gttggtgttgggagcgacgttgccgaagc
K525R	ctgatcgggctgctgcggactgcgcggctg	cagccgcgcagtccgcagcagcccgatcag
K525C	ctgatcgggctgctgtgtactgcgcggctgctg	cagcagccgcgcagtacacagcagcccgatcag
K525S	gagctgatcgggctgctgagtactgcgcggctg	cagccgcgcagtactcagcagcccgatcagctc
K525M	gatcgggctgctgatgactgcgcggc	gccgcgcagtcatcagcagcccgatc
K525P	gatcgggctgctgccgactgcgcggctg	cagccgcgcagtcggcagcagcccgatc
K525A	gatcgggctgctggcgactgcgcggctg	cagccgcgcagtcgccagcagcccgatc
K525V	gatcgggctgctggtgactgcgcggctg	cagccgcgcagtcaccagcagcccgatc
K525E	ctgatcgggctgctggagactgcgcgg	gccgcgcagtctccagcagcccgatcag

### Cell culture and transient transfection

Chinese Hamster Ovary (CHO) cells were grown in 50/50 DMEM/F12 (Cellgro, Manassas, VA) with 10% fetal bovine serum (FBS) and 2 mM L-glutamine (Gibco, Carlsbad, CA). To express ion channels, cells were split 24 hours before transfection, plated in 60-mm dishes, and transfected with Lipofectamine 3000™ reagent (Invitrogen, Carlsbad, CA), according to the manufacturer’s instruction. At 24 hours after transfection, cells were split and re-plated onto coverslips coated with poly-L-lysine (Sigma-Aldrich, St. Louis, MO). A plasmid for cDNA of GFP (Amaxa, Gaithersburg, MD) was co-transfected to aid the identification of transfected cells by fluorescence microscopy.

### Electrophysiological recording

Whole-cell voltage-clamp recording was carried out at room temperature in CHO cells with an Axopatch-200B amplifier (Molecular Devices, Sunnyvale, CA). The electrodes were pulled from borosilicate glass capillaries (World Precision Instruments, Sarasota, FL). When filled with the intracellular solution, the electrodes had resistances of 3-5 MΩ. The pipette solution contained (mM): KCl 145, MgCl_2_ 1, EGTA 5, HEPES 10, and MgATP 5 (pH 7.3 with KOH). During the recording, constant perfusion of the extracellular solution was maintained using a BPS perfusion system (ALA Scientific Instruments, Westbury, NY). The extracellular solution contained (mM): NaCl 140, KCl 5, CaCl_2_ 2, MgCl_2_ 1.5, HEPES 10, and glucose 10 (pH 7.4 with NaOH). Signals were filtered at 1 KHz and digitized using a DigiData 1550B with pClamp 11.2 software (Molecular Devices, Sunnyvale, CA). Series resistance was compensated by 60-80%.

### Determination of relative permeability (*P*_X_/*P*_K_)

The permeability of TMEM175 for Na^+^ and NMDG^+^ relative to K^+^ was estimated based on the Goldmann-Hodgkin-Katz current equation s[65] and the measured Erev using “bi-ionic” conditions:

$$\frac{P_X}{P_K}=\frac{{\left[{K}^{+}\right]}_i{e}^{\left(^{ FV}\!\left/ \!_{ RT}\right.\right)}-{\left[{K}^{+}\right]}_e}{{\left[{X}^{+}\right]}_e-{\left[{X}^{+}\right]}_i{e}^{\left(^{ FV}\!\left/ \!_{ RT}\right.\right)}}$$

Where R, T, F, and V are the gas constant, absolute temperature, Faraday`s constant, and the reversal potential, respectively. In the whole-cell recording, the pipette solution is (in mM): KCl 145, EGTA 5, and HEPES 10 (pH 7.3 with KOH). The sodium solution is (in mM): NaCl 145, EGTA 5, and HEPES 10 (pH 7.3 with NaOH). The NMDG solution is (in mM): NMDG 145, EGTA 5, and HEPES 10 (pH 7.3 with HCl). *E*rev was determined using a pre-open at 0 mV step protocol in response to extracellular solutions.

### Data and statistical analysis

Patch-clamp data were processed using Clampfit 11.2 (Molecular Devices, Sunnyvale, CA), and then analyzed in Origin 2018 (OriginLab, Northampton, MA). Voltage-dependent activation curves were fit with the Boltzmann equation: *G*=*G*_min_+(*G*_max_-*G*_min_)/(1+exp(*V*-*V*_1/2_)/S)), where *G*_max_ is the maximum conductance, *G*_min_ is the minimum conductance, *V*_1/2_ is the voltage for reaching 50% of the maximum conductance and S is the slope factor. Data are presented as means ± SEM. Significance was estimated using paired two-tailed students’ *t-*tests.

## Supplementary Information

**Additional file 1: Fig. S1.** K525N&G628S is a hyperpolarization-activated channel. **a** The expression pattern of hERG-EGFP, and F627Y-EGFP in CHO cells stained with Dil dye (red). **b** The representative ramp current of K525N&F627Y. The ramp protocol (below) ranges from -130 to +30 mV in 5 seconds with a holding potential at -100 mV. **c** Representative currents of G628S. **d** Representative currents of K525N&G628S. **e** The *G*-*V* and **e** the I-V curve of K525N&G628S.

**Additional file 2: Fig. S2.** K525N&G628S is a K^+^ selective channel. **a** Representative current traces with ramp protocols and **b** histogram of the reversal potential of K525N&F627Y in the presence of different bath solutions. **c** Representative current traces and **d** histogram of the reversal potential of WT hERG channel in the presence of different bath solutions. **e** Representative current traces and **f** histogram of the reversal potential of K525N&G628S in the presence of different bath solutions. **g** Analysis of relative K^+^ permeability over Na^+^, or NMDG^+^ of K525N&G628S based on *E*rev measurement (n≥6). Error bars represent mean ± SEM.

**Additional file 3: Fig. S3.** Various K525 mutations disturb channel closure. **a** *G*-*V* curves of K525 mutants.

**Additional file 4: Fig.** **4.** I419A did not impair the inward current of K525N&F627Y. **a** The expression pattern of K525N&F627Y&F431A-EGFP, K525N&F627Y&C566A-EGFP, K525N&F627Y&I607A-EGFP, and K525N&F627Y&Y611A-EGFP in CHO cells stained with Dil dye (red). **b** The cryo-EM structure of hERG (PDB: 5VA1) highlighted with K525 (blue), F627 (yellow), and F431 (red) and I419 (green) residues amongst the potential interaction face between the VSD and PD. The top view and side view of the interaction region are zoomed out and shown in the *Right* panel. **c** Representative step currents of K525N&F627Y, and K525N&F627Y&I419A, respectively. **d** Current density (n≥6) of K525N&F627Y, and K525N&F627Y&I419A, respectively. Representative currents of **e** K525N, **f** K525N&F431A, **g** K525N&C566A, and **h** K525N&Y611A. Error bars represent mean ± SEM. **P<0.01; ***P<0.001, two-tailed Student’s t-test.

## Data Availability

All data generated or analyzed during this study are included in this published article and its supplementary information files.

## Abbreviations

CNBD: Cyclic nucleotide-binding domain
hERG: *human Ether-a-go-go Related Gene*
VSD: Voltage-sensing domain
LQT2: Long-QT syndrome type 2
PD: Pore domain
*I*_Kr_: Rapidly activating delayed rectifier potassium current
ECG: Electrocardiogram
Kv: Voltage-gated potassium channel
HCN: Hyperpolarization-activated and cyclic nucleotide-gated
CHO: Chinese Hamster Ovary
WT: Wild-type

## References

[1] Vandenberg JI, Perry MD, Perrin MJ, Mann SA, Ke Y, Hill AP. hERG K(+) channels: structure, function, and clinical significance. Physiol Rev. 2012;92(3):1393–478. 22988594 10.1152/physrev.00036.2011

[2] Bohnen MS, Peng G, Robey SH, Terrenoire C, Iyer V, Sampson KJ, Kass RS. Molecular Pathophysiology of Congenital Long QT Syndrome. Physiol Rev. 2017;97(1):89–134. 27807201 10.1152/physrev.00008.2016 PMC5539372

[3] Smith JL, Anderson CL, Burgess DE, Elayi CS, January CT, Delisle BP. Molecular pathogenesis of long QT syndrome type 2. J Arrhythm. 2016;32(5):373–80. 27761161 10.1016/j.joa.2015.11.009 PMC5063260

[4] Li J, Shen R, Reddy B, Perozo E, Roux B. Mechanism of C-type inactivation in the hERG potassium channel. Sci Adv. 2021;7(5). 10.1126/sciadv.abd6203 PMC7846155 33514547

[5] Miranda WE, DeMarco KR, Guo J, Duff HJ, Vorobyov I, Clancy CE, Noskov SY. Selectivity filter modalities and rapid inactivation of the hERG1 channel. Proc Natl Acad Sci U S A. 2020;117(6):2795–804. 31980532 10.1073/pnas.1909196117 PMC7022143

[6] Wang W, MacKinnon R. Cryo-EM Structure of the Open Human Ether-a-go-go-Related K(+) Channel hERG. Cell. 2017;169(3):422-430 e410. 28431243 10.1016/j.cell.2017.03.048 PMC5484391

[7] James ZM, Zagotta WN. Structural insights into the mechanisms of CNBD channel function. J Gen Physiol. 2018;150(2):225–44. 29233886 10.1085/jgp.201711898 PMC5806680

[8] Lee CH, MacKinnon R. Voltage Sensor Movements during Hyperpolarization in the HCN Channel. Cell. 2019;179(7):1582-1589 e1587. 31787376 10.1016/j.cell.2019.11.006 PMC6911011

[9] Lee CH, MacKinnon R. Structures of the Human HCN1 Hyperpolarization-Activated Channel. Cell. 2017;168(1–2):111-120 e111. 28086084 10.1016/j.cell.2016.12.023 PMC5496774

[10] Piper DR, Sanguinetti MC, Tristani-Firouzi M. Voltage sensor movement in the hERG K+ channel. Novartis Found Symp. 2005;266:46–52 discussion 52-46, 95-49. 16050261 10.1002/047002142X.ch5

[11] Cheng YM, Claydon TW. Voltage-dependent gating of HERG potassium channels. Front Pharmacol. 2012;3:83. 22586397 10.3389/fphar.2012.00083 PMC3347040

[12] Tombola F, Pathak MM, Isacoff EY. How does voltage open an ion channel? Annu Rev Cell Dev Biol. 2006;22:23–52. 16704338 10.1146/annurev.cellbio.21.020404.145837

[13] Jensen MO, Jogini V, Borhani DW, Leffler AE, Dror RO, Shaw DE. Mechanism of voltage gating in potassium channels. Science. 2012;336(6078):229–33. 22499946 10.1126/science.1216533

[14] Zhang M, Liu J, Tseng GN. Gating charges in the activation and inactivation processes of the HERG channel. J Gen Physiol. 2004;124(6):703–18. 15545400 10.1085/jgp.200409119 PMC2234031

[15] Smith PL, Yellen G. Fast and slow voltage sensor movements in HERG potassium channels. J Gen Physiol. 2002;119(3):275–93. 11865022 10.1085/jgp.20028534 PMC2217288

[16] Van Slyke AC, Rezazadeh S, Snopkowski M, Shi P, Allard CR, Claydon TW. Mutations within the S4-S5 linker alter voltage sensor constraints in hERG K+ channels. Biophys J. 2010;99(9):2841–52. 21044581 10.1016/j.bpj.2010.08.030 PMC2965951

[17] Flynn GE, Zagotta WN. Insights into the molecular mechanism for hyperpolarization-dependent activation of HCN channels. Proc Natl Acad Sci U S A. 2018;115(34):E8086–95. 30076228 10.1073/pnas.1805596115 PMC6112743

[18] Lee SY, Banerjee A, MacKinnon R. Two separate interfaces between the voltage sensor and pore are required for the function of voltage-dependent K(+) channels. PLoS Biol. 2009;7(3):e47. 19260762 10.1371/journal.pbio.1000047 PMC2650729

[19] Phan K, Ng CA, David E, Shishmarev D, Kuchel PW, Vandenberg JI, Perry MD. The S1 helix critically regulates the finely tuned gating of Kv11.1 channels. J Biol Chem. 2017;292(18):7688–705. 28280240 10.1074/jbc.M117.779298 PMC5418064

[20] Bassetto CA, Carvalho-de-Souza JL, Bezanilla F. Molecular basis for functional connectivity between the voltage sensor and the selectivity filter gate in Shaker K(+) channels. Elife. 2021;10. 10.7554/eLife.63077 PMC7943188 33620313

[21] Vardanyan V, Pongs O. Coupling of voltage-sensors to the channel pore: a comparative view. Front Pharmacol. 2012;3:145. 22866036 10.3389/fphar.2012.00145 PMC3406610

[22] Colenso CK, Sessions RB, Zhang YH, Hancox JC, Dempsey CE. Interactions between Voltage Sensor and Pore Domains in a hERG K Channel Model from Molecular Simulations and the Effects of a Voltage Sensor Mutation. J Chem Inf Model. 2013;53. 10.1021/ci4000739 23672495

[23] Tristani-Firouzi M, Chen J, Sanguinetti MC. Interactions between S4-S5 linker and S6 transmembrane domain modulate gating of HERG K+ channels. J Biol Chem. 2002;277(21):18994–9000. 11864984 10.1074/jbc.M200410200

[24] Liu J, Zhang M, Jiang M, Tseng GN. Structural and functional role of the extracellular s5-p linker in the HERG potassium channel. J Gen Physiol. 2002;120(5):723–37. 12407082 10.1085/jgp.20028687 PMC2229555

[25] Bassetto CAZ Jr, Costa F, Guardiani C, Bezanilla F, Giacomello A. Noncanonical electromechanical coupling paths in cardiac hERG potassium channel. Nat Commun. 2023;14(1):1110. 36849440 10.1038/s41467-023-36730-7 PMC9971164

[26] Sanguinetti MC, Xu QP. Mutations of the S4-S5 linker alter activation properties of HERG potassium channels expressed in Xenopus oocytes. J Physiol. 1999;514(Pt 3):667–75. 9882738 10.1111/j.1469-7793.1999.667ad.x PMC2269111

[27] Thouta S, Sokolov S, Abe Y, Clark SJ, Cheng YM, Claydon TW. Proline scan of the HERG channel S6 helix reveals the location of the intracellular pore gate. Biophys J. 2014;106(5):1057–69. 24606930 10.1016/j.bpj.2014.01.035 PMC4026777

[28] Zhao Y, Scheuer T, Catterall WA. Reversed voltage-dependent gating of a bacterial sodium channel with proline substitutions in the S6 transmembrane segment. Proc Natl Acad Sci U S A. 2004;101(51):17873–8. 15583130 10.1073/pnas.0408270101 PMC539779

[29] Li L, Liu K, Hu Y, Li D, Luan S. Single mutations convert an outward K+ channel into an inward K+ channel. Proc Natl Acad Sci U S A. 2008;105(8):2871–6. 18287042 10.1073/pnas.0712349105 PMC2268552

[30] Millat G, Chevalier P, Restier-Miron L, Da Costa A, Bouvagnet P, Kugener B, Fayol L, Gonzalez Armengod C, Oddou B, Chanavat V, et al. Spectrum of pathogenic mutations and associated polymorphisms in a cohort of 44 unrelated patients with long QT syndrome. Clin Genet. 2006;70(3):214–27. 16922724 10.1111/j.1399-0004.2006.00671.x

[31] Pettini F, Domene C, Furini S. Early Steps in C-Type Inactivation of the hERG Potassium Channel. J Chem Inf Model. 2023;63(1):251–8. 36512342 10.1021/acs.jcim.2c01028 PMC9832476

[32] Gang H, Zhang S. Na+ permeation and block of hERG potassium channels. J Gen Physiol. 2006;128(1):55–71. 16769794 10.1085/jgp.200609500 PMC2151557

[33] Zhao Y, Wang T, Guo J, Yang T, Li W, Koichopolos J, Lamothe SM, Kang Y, Ma A, Zhang S. Febrile temperature facilitates hERG/IKr degradation through an altered K(+) dependence. Heart Rhythm. 2016;13(10):2004–11. 27321242 10.1016/j.hrthm.2016.06.019

[34] Coonen L, Martinez-Morales E, Van De Sande DV, Snyders DJ, Cortes DM, Cuello LG, Labro AJ. The nonconducting W434F mutant adopts upon membrane depolarization an inactivated-like state that differs from wild-type Shaker-IR potassium channels. Sci Adv. 2022;8(37):eabn1731. 36112676 10.1126/sciadv.abn1731 PMC9481120

[35] Jurkat-Rott K, Groome J, Lehmann-Horn F. Pathophysiological role of omega pore current in channelopathies. Front Pharmacol. 2012;3:112. 22701429 10.3389/fphar.2012.00112 PMC3372090

[36] Jiang D, Gamal El-Din TM, Ing C, Lu P, Pomes R, Zheng N, Catterall WA. Structural basis for gating pore current in periodic paralysis. Nature. 2018;557(7706):590–4. 29769724 10.1038/s41586-018-0120-4 PMC6708612

[37] Moreau A, Gosselin-Badaroudine P, Chahine M. Biophysics, pathophysiology, and pharmacology of ion channel gating pores. Front Pharmacol. 2014;5:53. 24772081 10.3389/fphar.2014.00053 PMC3982104

[38] Kudaibergenova M, Guo J, Khan HM, Lees-Miller J, Mousaei M, Miranda W, Ngo VA, Noskov SY, Tieleman DP, Duff HJ. The voltage-sensing domain of a hERG1 mutant is a cation-selective channel. Biophysical J. 2022;121(23):14. 10.1016/j.bpj.2022.10.032 PMC9748372 36815709

[39] Perry M, Sanguinetti M, Mitcheson J. Revealing the structural basis of action of hERG potassium channel activators and blockers. J Physiol. 2010;588(Pt 17):3157–67. 20643767 10.1113/jphysiol.2010.194670 PMC2976011

[40] Kamiya K, Niwa R, Mitcheson JS, Sanguinetti MC. Molecular determinants of HERG channel block. Mol Pharmacol. 2006;69(5):1709–16. 16474003 10.1124/mol.105.020990

[41] Lees-Miller JP, Duan Y, Teng GQ, Duff HJ. Molecular determinant of high-affinity dofetilide binding to HERG1 expressed in Xenopus oocytes: involvement of S6 sites. Mol Pharmacol. 2000;57(2):367–74. 10648647

[42] Choveau FS, El Harchi A, Rodriguez N, Louerat-Oriou B, Baro I, Demolombe S, Charpentier F, Loussouarn G. Transfer of rolf S3-S4 linker to HERG eliminates activation gating but spares inactivation. Biophys J. 2009;97(5):1323–34. 19720020 10.1016/j.bpj.2009.05.060 PMC2749759

[43] Dou Y, Goodchild SJ, Velde RV, Wu Y, Fedida D. The neutral, hydrophobic isoleucine at position I521 in the extracellular S4 domain of hERG contributes to channel gating equilibrium. Am J Physiol Cell Physiol. 2013;305(4):C468–78. 23761630 10.1152/ajpcell.00147.2013

[44] Kamiya K, Niwa R, Morishima M, Honjo H, Sanguinetti MC. Molecular determinants of hERG channel block by terfenadine and cisapride. J Pharmacol Sci. 2008;108(3):301–7. 18987434 10.1254/jphs.08102FP PMC2845965

[45] McPate MJ, Duncan RS, Witchel HJ, Hancox JC. Disopyramide is an effective inhibitor of mutant HERG K+ channels involved in variant 1 short QT syndrome. J Mol Cell Cardiol. 2006;41(3):563–6. 16842817 10.1016/j.yjmcc.2006.05.021

[46] Walker BD, Singleton CB, Bursill JA, Wyse KR, Valenzuela SM, Qiu MR, Breit SN, Campbell TJ. Inhibition of the human ether-a-go-go-related gene (HERG) potassium channel by cisapride: affinity for open and inactivated states. Br J Pharmacol. 1999;128(2):444–50. 10510456 10.1038/sj.bjp.0702774 PMC1571630

[47] Butler A, Helliwell MV, Zhang Y, Hancox JC, Dempsey CE. An Update on the Structure of hERG. Front Pharmacol. 2019;10:1572. 32038248 10.3389/fphar.2019.01572 PMC6992539

[48] Zou A, Xu QP, Sanguinetti MC. A mutation in the pore region of HERG K+ channels expressed in Xenopus oocytes reduces rectification by shifting the voltage dependence of inactivation. J Physiol. 1998;509(Pt 1):129–37. 9547387 10.1111/j.1469-7793.1998.129bo.x PMC2230942

[49] Schonherr R, Heinemann SH. Molecular determinants for activation and inactivation of HERG, a human inward rectifier potassium channel. J Physiol. 1996;493(Pt 3):635–42. 8799887 10.1113/jphysiol.1996.sp021410 PMC1159013

[50] Gerlach AC, Stoehr SJ, Castle NA. Pharmacological removal of human ether-a-go-go-related gene potassium channel inactivation by 3-nitro-N-(4-phenoxyphenyl) benzamide (ICA-105574). Mol Pharmacol. 2010;77(1):58–68. 19805508 10.1124/mol.109.059543

[51] Zangerl-Plessl EM, Berger M, Drescher M, Chen Y, Wu W, Maulide N, Sanguinetti M, Stary-Weinzinger A. Toward a Structural View of hERG Activation by the Small-Molecule Activator ICA-105574. J Chem Inf Model. 2020;60(1):360–71. 31877041 10.1021/acs.jcim.9b00737

[52] Garg V, Stary-Weinzinger A, Sachse F, Sanguinetti MC. Molecular determinants for activation of human ether-a-go-go-related gene 1 potassium channels by 3-nitro-n-(4-phenoxyphenyl) benzamide. Mol Pharmacol. 2011;80(4):630–7. 21743002 10.1124/mol.111.073809 PMC3187531

[53] Kasimova MA, Tewari D, Cowgill JB, Ursuleaz WC, Lin JL, Delemotte L, et al. Helix breaking transition in the S4 of HCN channel is critical for hyperpolarization-dependent gating. Elife. 2019;8. 10.7554/eLife.53400 PMC6904216 31774399

[54] Cheng YM, Hull CM, Niven CM, Qi J, Allard CR, Claydon TW. Functional interactions of voltage sensor charges with an S2 hydrophobic plug in hERG channels. J Gen Physiol. 2013;142(3):289–303. 23980197 10.1085/jgp.201310992 PMC3753600

[55] Subbiah RN, Kondo M, Campbell TJ, Vandenberg JI. Tryptophan scanning mutagenesis of the HERG K+ channel: the S4 domain is loosely packed and likely to be lipid exposed. J Physiol. 2005;569(Pt 2):367–79. 16166152 10.1113/jphysiol.2005.097386 PMC1464230

[56] Zhang M, Liu J, Jiang M, Wu DM, Sonawane K, Guy HR, Tseng GN. Interactions between charged residues in the transmembrane segments of the voltage-sensing domain in the hERG channel. J Membr Biol. 2005;207(3):169–81. 16550488 10.1007/s00232-005-0812-1

[57] Guo J, Cheng YM, Lees-Miller JP, Perissinotti LL, Claydon TW, Hull CM, Thouta S, Roach DE, Durdagi S, Noskov SY, Duff HJ. NS1643 interacts around L529 of hERG to alter voltage sensor movement on the path to activation. Biophys J. 2015;108(6):1400–13. 25809253 10.1016/j.bpj.2014.12.055 PMC4375528

[58] Gardner A, Wu W, Thomson S, Zangerl-Plessl EM, Stary-Weinzinger A, Sanguinetti MC. Molecular Basis of Altered hERG1 Channel Gating Induced by Ginsenoside Rg3. Mol Pharmacol. 2017;92(4):437–50. 28705808 10.1124/mol.117.108886 PMC5588553

[59] Panaghie G, Abbott GW. The role of S4 charges in voltage-dependent and voltage-independent KCNQ1 potassium channel complexes. J Gen Physiol. 2007;129(2):121–33. 17227916 10.1085/jgp.200609612 PMC2154355

[60] Posson DJ, McCoy JG, Nimigean CM. The voltage-dependent gate in MthK potassium channels is located at the selectivity filter. Nat Struct Mol Biol. 2013;20(2):159–66. 23262489 10.1038/nsmb.2473 PMC3565016

[61] Kapplinger JD, Tester DJ, Salisbury BA, Carr JL, Harris-Kerr C, Pollevick GD, Wilde AA, Ackerman MJ. Spectrum and prevalence of mutations from the first 2,500 consecutive unrelated patients referred for the FAMILION long QT syndrome genetic test. Heart Rhythm. 2009;6(9):1297–303. 19716085 10.1016/j.hrthm.2009.05.021 PMC3049907

[62] Anderson CL, Kuzmicki CE, Childs RR, Hintz CJ, Delisle BP, January CT. Large-scale mutational analysis of Kv11.1 reveals molecular insights into type 2 long QT syndrome. Nat Commun. 2014;5:5535. 25417810 10.1038/ncomms6535 PMC4243539

[63] Ficker E, Obejero-Paz CA, Zhao S, Brown AM. The binding site for channel blockers that rescue misprocessed human long QT syndrome type 2 ether-a-gogo-related gene (HERG) mutations. J Biol Chem. 2002;277(7):4989–98. 11741928 10.1074/jbc.M107345200

[64] Li P, Chen X, Zhang Q, Zheng Y, Jiang H, Yang H, Gao Z. The human ether-a-go-go-related gene activator NS1643 enhances epilepsy-associated KCNQ channels. J Pharmacol Exp Ther. 2014;351(3):596–604. 25232191 10.1124/jpet.114.217703

[65] Lewis CA. Ion-concentration dependence of the reversal potential and the single channel conductance of ion channels at the frog neuromuscular junction. J Physiol. 1979;286:417–45. 312319 10.1113/jphysiol.1979.sp012629 PMC1281581

