# Supplementary figures and images for: An LQT2-related mutation in the voltage-sensing domain is involved in switching the gating polarity of hERG
Source: BMC Biol. 2024 Feb 5;22:29. doi: 10.1186/s12915-024-01833-0 (PMC11380439; doi:10.1186/s12915-024-01833-0)

**a**

CHO

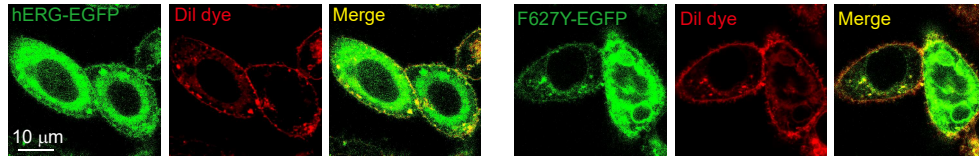

**b**

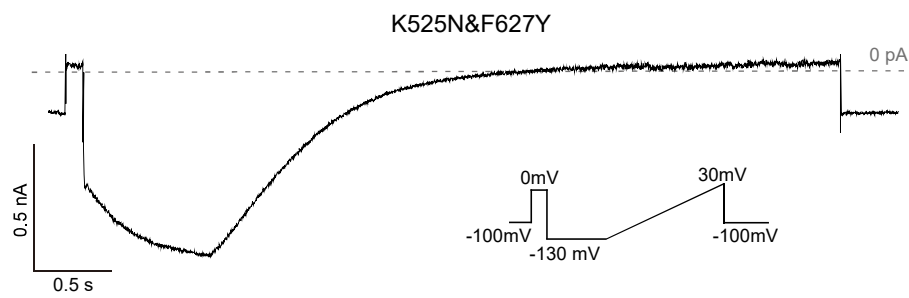

**c**

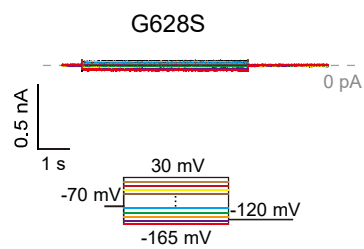

**d**

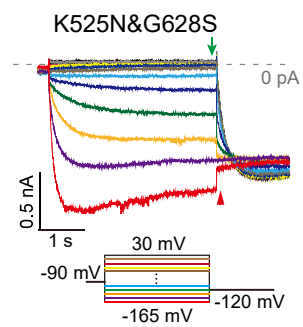

**e**

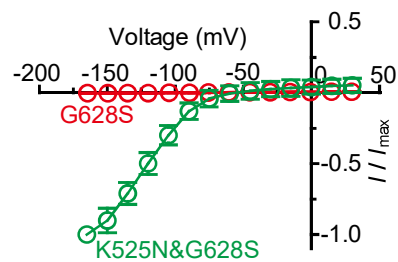

**f**

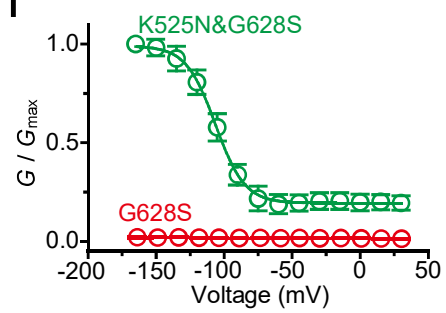

*Liu et al., Additional file 1*

Supplement: Supplementary file 1 — Additional file 1: Fig. S1. K525N&G628S is a hyperpolarization-activated channel. a The expression pattern of hERG-EGFP, and F627Y-EGFP in CHO cells stained with Dil dye (red). b The representative ramp current of K525N&F627Y. The ramp protocol (below) ranges from -130 to +30 mV in 5 seconds with a holding potential at -100 mV. c Representative currents of G628S. d Representative currents of K525N&G628S. e The G-V and e the I-V curve of K525N&G628S. [file 12915_2024_1833_MOESM1_ESM.pdf]

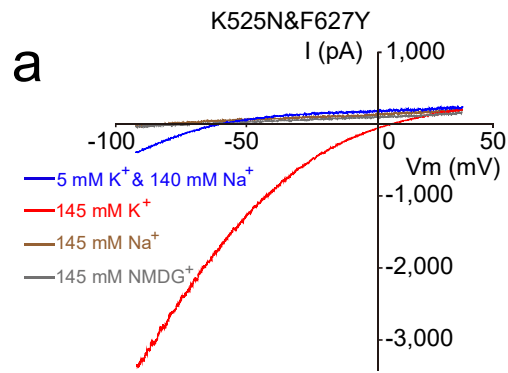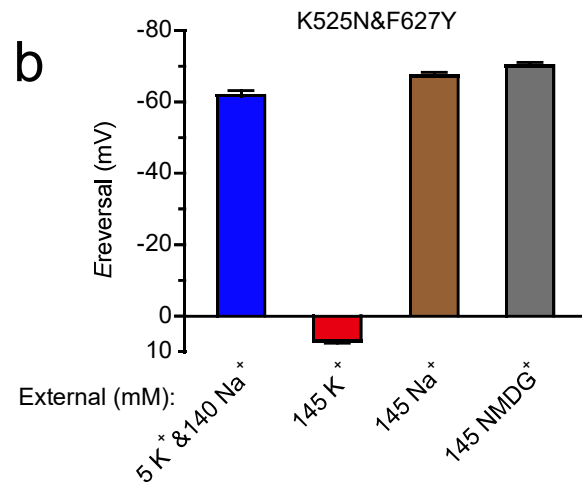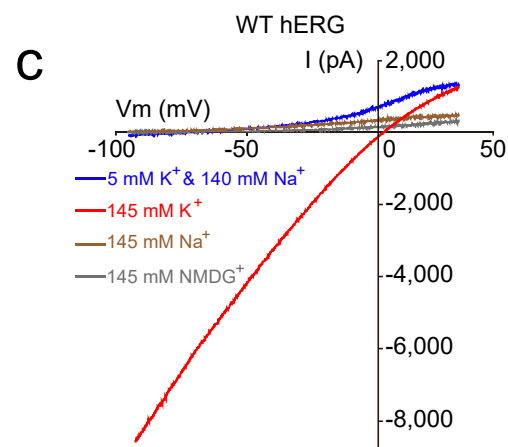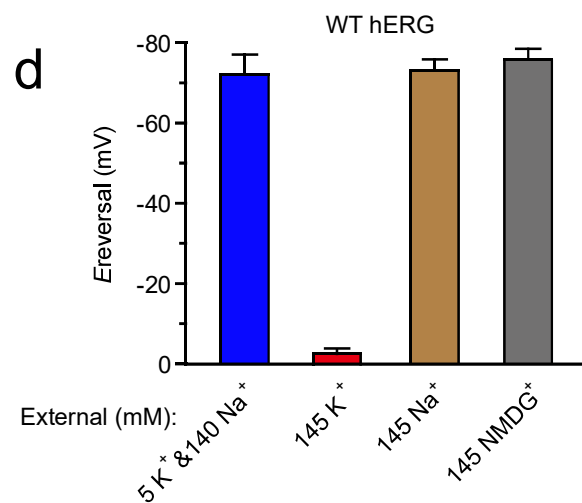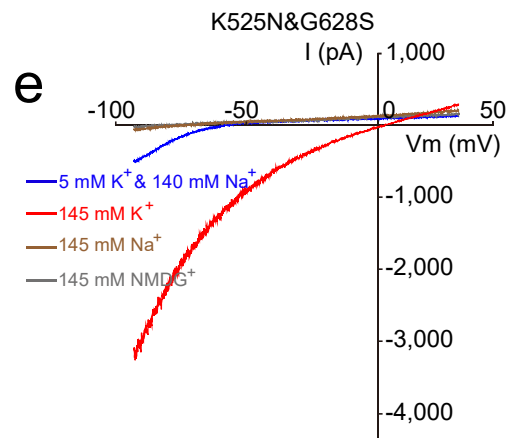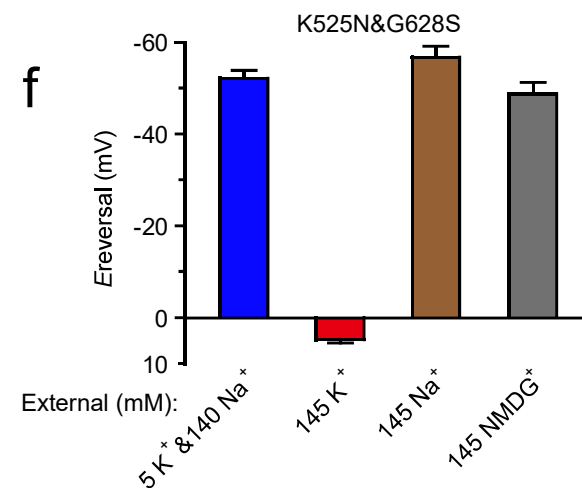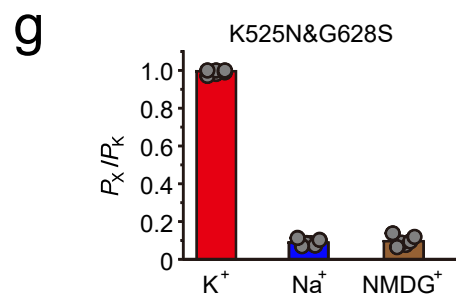

Supplement: Supplementary file 2 — Additional file 2: Fig. S2. K525N&G628S is a K+ selective channel. a Representative current traces with ramp protocols and b histogram of the reversal potential of K525N&F627Y in the presence of different bath solutions. c Representative current traces and d histogram of the reversal potential of WT hERG channel in the presence of different bath solutions. e Representative current traces and f histogram of the reversal potential of K525N&G628S in the presence of different bath solutions. g Analysis of relative K+ permeability over Na+, or NMDG+ of K525N&G628S based on Erev measurement (n≥6). Error bars represent mean ± SEM. [file 12915_2024_1833_MOESM2_ESM.pdf]

a

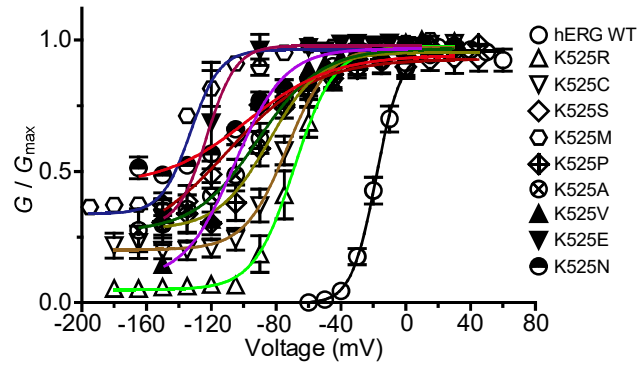

*Liu et al., Additional file 3*

Supplement: Supplementary file 3 — Additional file 3: Fig. S3. Various K525 mutations disturb channel closure. a G-V curves of K525 mutants. [file 12915_2024_1833_MOESM3_ESM.pdf]

**a**

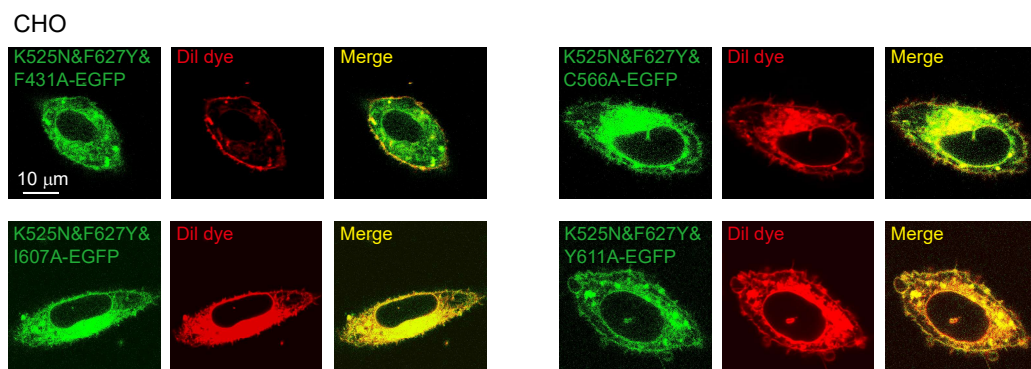

**b**

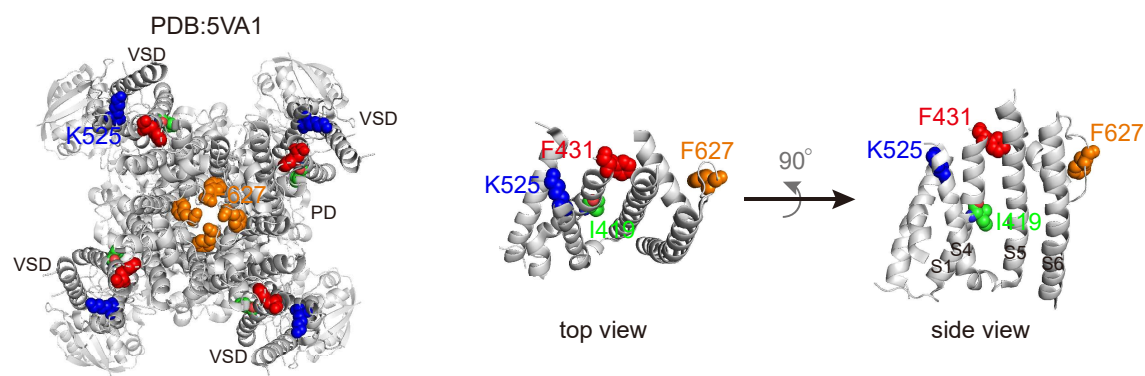

**c**

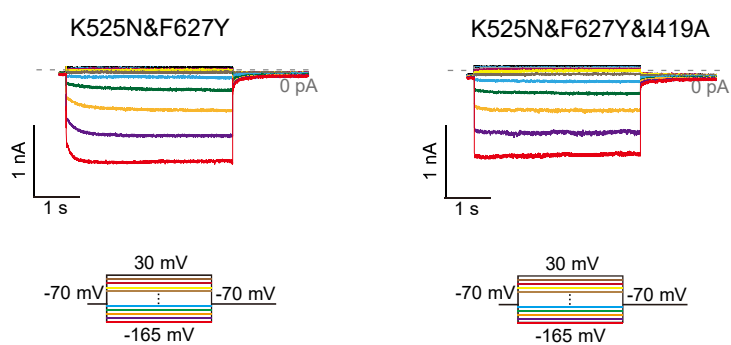

**d**

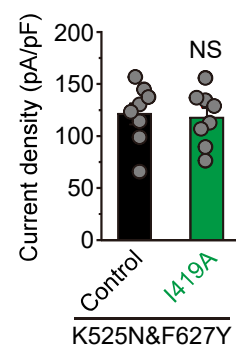

**e**

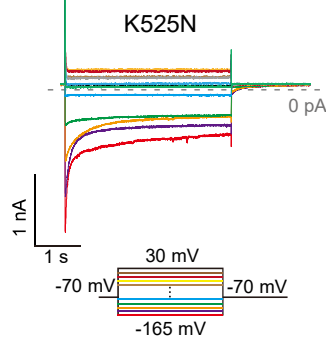

**f**

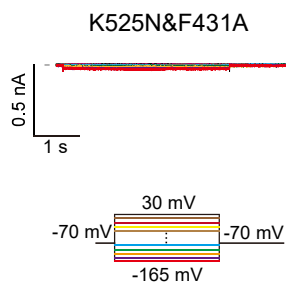

**g**

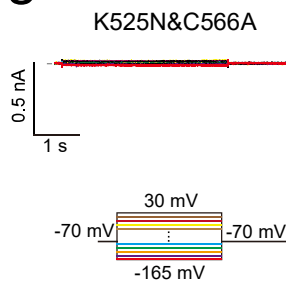

**h**

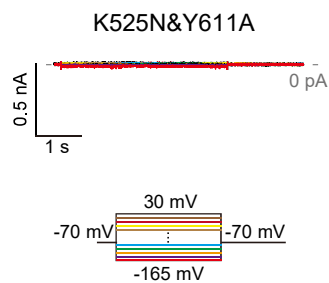

Supplement: Supplementary file 4 — Additional file 4: Fig. 4. I419A did not impair the inward current of K525N&F627Y. a The expression pattern of K525N&F627Y&F431A-EGFP, K525N&F627Y&C566A-EGFP, K525N&F627Y&I607A-EGFP, and K525N&F627Y&Y611A-EGFP in CHO cells stained with Dil dye (red). b The cryo-EM structure of hERG (PDB: 5VA1) highlighted with K525 (blue), F627 (yellow), and F431 (red) and I419 (green) residues amongst the potential interaction face between the VSD and PD. The top view and side view of the interaction region are zoomed out and shown in the Right panel. c Representative step currents of K525N&F627Y, and K525N&F627Y&I419A, respectively. d Current density (n≥6) of K525N&F627Y, and K525N&F627Y&I419A, respectively. Representative currents of e K525N, f K525N&F431A, g K525N&C566A, and h K525N&Y611A. Error bars represent mean ± SEM. **P<0.01; ***P<0.001, two-tailed Student’s t-test. [file 12915_2024_1833_MOESM4_ESM.pdf]
